# Circulating Tumor DNA (ctDNA) in Gastroesophageal Adenocarcinoma (GEA): Evidence and Emerging Applications

**DOI:** 10.3390/cancers17223692

**Published:** 2025-11-18

**Authors:** Oudai Sahwan, Lin Batha, Fares Jamal, Mohamad Bassam Sonbol

**Affiliations:** 1 Division of Hematology/Oncology, Comprehensive Cancer Center, Mayo Clinic, Phoenix, AZ 85054, USAsonbol.mohamad@mayo.edu (M.B.S.); 2 College of Medicine, Alfaisal University, Riyadh 11533, Saudi Arabia; lbatha@alfaisal.edu

**Keywords:** circulating tumor DNA (ctDNA), gastroesophageal adenocarcinoma, liquid biopsy, minimal residual disease, tumor-informed sequencing, biomarker-guided therapy

## Abstract

The role of circulating tumor DNA (ctDNA) in gastroesophageal adenocarcinoma (GEA) has expanded in recent years. In resectable disease, postoperative ctDNA is able to detect patients at highest risk of recurrence months before scans. Tumor-informed assays provide the best sensitivity and emerging methylation assays are useful when tissue is scarce. In metastatic GEA, baseline ctDNA burden correlates with prognosis, and a decrease in ctDNA level after treatment initiation reflects therapeutic response. It can also uncover actionable targets, including *ERBB2*, *FGFR2*, and MSI-H, and detect resistance that can arise after starting treatment. Limitations include variable assay performance, low shedding in some tumors, clonal hematopoiesis confounding, and a lack of randomized data showing that ctDNA-guided changes improve outcomes. Ongoing trials are testing MRD-guided escalation/de-escalation and ctDNA-directed biomarker therapy. In this review, we evaluate the role of ctDNA in GEA cancers over recent years.

## Simple Summary

Circulating tumor DNA (ctDNA) has emerged as a promising non-invasive biomarker that can be detected in the blood of patients with gastroesophageal adenocarcinoma (GEA). Measuring ctDNA levels can help identify patients at high risk of recurrence after surgery, evaluate treatment response, and detect molecular changes that signal resistance to therapy. ctDNA can also uncover actionable biomarkers such as ERBB2 or MSI-H and reveal resistance mechanisms. In this review, we summarize recent studies on the clinical applications of ctDNA in resectable and advanced GEA.

## 1. Introduction

Gastroesophageal adenocarcinoma (GEA), encompassing adenocarcinomas arising from the esophagus, esophagogastric junction and stomach, possesses a significant global health burden. In 2022, gastric cancer ranked as the fifth most common cancer worldwide with 968,350 new cases and 659,853 deaths [1]. Its incidence is projected to reach 1.77 million cases by 2040 [2]. In the United States alone, gastric cancer accounted for 30,300 new cases and 10,780 deaths from the disease [3]. Although early-stage esophageal and gastric adenocarcinomas have relatively favorable prognoses, most patients present with advanced disease, leading to worse survival [4,5].

Management approaches for GEA vary greatly based on the disease stage at diagnosis. For early-stage disease (stage I–II), the mainstay of management is curative-intent surgery, often in combination with perioperative systemic therapy, or neoadjuvant chemoradiotherapy [6,7,8,9,10,11]. For advanced and/or metastatic GEA, systemic therapy is required, typically involving chemotherapy, targeted therapies, and/or immunotherapy [11,12,13,14,15,16,17,18,19,20]. Additionally, treatment choice relies on different molecular biomarkers, including human epidermal growth factor receptor 2 (HER2) amplification, mismatch repair deficient (dMMR)/microsatellite instability-high (MSI-H), programmed death ligand 1 (PD-L1), Claudin 18.2, and in the near future, fibroblast growth factor receptor 2 (*FGFR2*) amplification, among others [21]. This points to the increasingly pivotal role biomarkers are playing in driving personalized therapeutic decisions. It is evident, therefore, that appropriate biomarker profiling is essential in maximizing treatment outcomes and optimizing clinical management in GEA.

Over the past decade, liquid biopsy (LB) has emerged as a promising and minimally invasive strategy for the management of cancer [22]. LB can detect and assess different types of biomarkers such as circulating tumor cells (CTCs), proteins, cell-free ribonucleic acid (cfRNA), autoantibodies, extracellular vesicles, and cell-free deoxyribonucleic acid (cfDNA) [23]. Among them, cfDNA has gained considerable popularity. Recent research has focused specifically on the tumor-derived component of cfDNA, known as circulating tumor DNA (ctDNA), which offers greater specificity for cancer detection and monitoring. Due to its close relationship with the tumor, ctDNA has emerged as the defining biomarker in LB and has been a trending area of research in recent years [24].

The applications of ctDNA include early detection and diagnosis, molecular profiling, monitoring for minimal residual disease (MRD), assessing treatment response, and identifying resistance mechanisms/actionable alterations [25]. Its clinical utility is well-established in several malignancies including colorectal, lung, and breast cancers [24,25,26,27]. This has paved the way for Food and Drug Administration (FDA)-approved technologies based on ctDNA (i.e., Guardant360 CDx and FoundationOne Liquid CDx) that allow for the real-time monitoring of cancer and genomic profiling [28,29]. In GEA, ctDNA as an MRD method has not reached routine clinical practice yet, but several studies have investigated its uses.

In this narrative review, we evaluated the evidence currently available for the use and limitations of ctDNA in the workup and management of GEA.

## 2. ctDNA

In healthy people, hematopoietic cells are the main source of cfDNA; however, in cancer patients, a variable fraction of cfDNA comes from tumor cells and is therefore termed ctDNA [30]. ctDNA, composed of DNA fragments ranging from 70 to 200 base pairs to 21 kb fragments, can be released into the systemic circulation by the primary/metastatic tumor cells, and CTCs via cell apoptosis, necrosis, or active secretion [31,32]. ctDNA fragments are typically smaller than normal cfDNA which helps in their detection [33]. The growing interest in ctDNA is owed to its theoretical advantages over conventional tissue biopsies, as it is less invasive, uses fewer resources, and makes it possible to capture changes in tumor heterogeneity, molecular evolution, and treatment response as it can be repeated over time [34,35]. Additionally, ctDNA levels are believed to correlate closely with the overall tumor burden, and numerous studies have demonstrated strong concordance between genetic alterations detected in tumor tissue (including copy number changes, single nucleotide variants, point mutations, and epigenetic modifications) and those identified in ctDNA; especially given that ctDNA is shed by the multiple tumor subclones within the same patient [35,36]. Notably, owing to the short half-life of ctDNA, ranging between 16 min and 2.5 h, ctDNA can reflect tumor dynamics in near real-time, making it a valuable tool for monitoring disease progression and serving as a potential prognostic biomarker [37,38].

**Figure 1:**
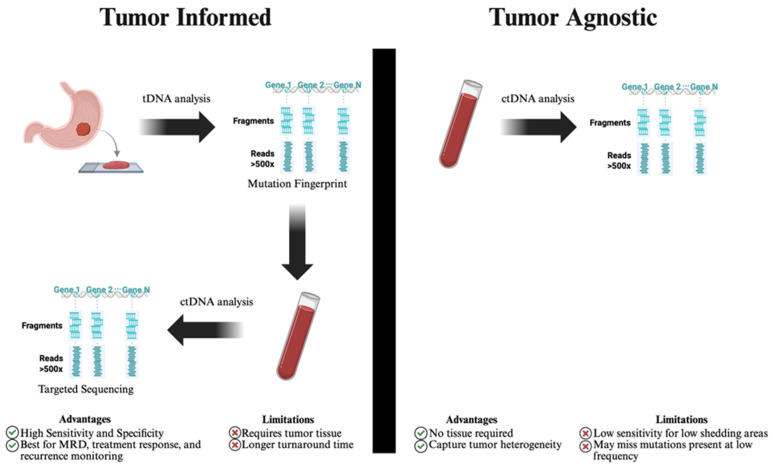
Comparison of tumor-informed and tumor-agnostic ctDNA testing approaches. ctDNA: circulating tumor deoxyribonucleic acid, MRD: minimal residual disease, tDNA: tissue deoxyribonucleic acid. Tumor-informed ctDNA analysis begins with tissue-based sequencing to identify a patient-specific mutation fingerprint, which is then tracked in plasma using targeted sequencing. This approach offers high sensitivity and is ideal for minimal residual disease (MRD) detection and treatment monitoring but requires tumor tissue and has longer turnaround time. Tumor-agnostic analysis, in contrast, directly sequences ctDNA from plasma without prior tumor profiling, enabling detection of actionable mutations and tumor heterogeneity, though it may have reduced sensitivity in low-shedding tumors or low-allele-frequency variants.

There are two general approaches to quantifying ctDNA, tumor-informed (targeted) and tumor-agnostic (untargeted) (Figure 1). The tumor-informed approach requires prior knowledge of the specific mutations or alterations contained within the tumor, which necessitates a tissue biopsy. This approach has repeatedly shown higher sensitivity and specificity and is therefore appropriate to assess for MRD, evaluate treatment response, and monitor early recurrence [39,40,41]. The tumor-agnostic approach, on the other hand, does not require prior tumor knowledge, which makes it cost-effective and accessible, especially when tumor tissue is unavailable. It enables the comprehensive characterization of the molecular landscape of the tumor and facilitates monitoring acquired mutations and resistance mechanisms, making it ideal to capture intratumor heterogeneity [39,42]. Importantly, tumor-agnostic tests have the benefit of faster turn-around times; however, they do come at the cost of lower sensitivity and requiring high concentrations of ctDNA [39]. Ultimately, the appropriate approach should be selected based on the clinical context. Figure 1 compares tumor-informed and tumor-agnostic ctDNA testing approaches.

## 3. MRD

### 3.1. Prognostic Value of ctDNA-Based MRD

**Figure 2:**
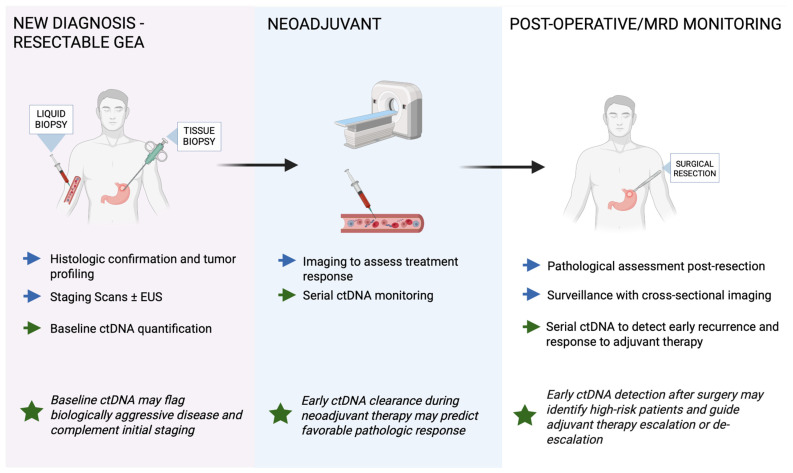
Clinical workflow of potential ctDNA applications in resectable gastroesophageal adenocarcinoma (GEA). Blue arrows indicate standard management steps, while green arrows represent ctDNA applications across diagnostic, neoadjuvant, and postoperative phases. Green stars highlight key clinical implications. ctDNA can complement standard tissue-based testing at diagnosis, provide dynamic monitoring during neoadjuvant therapy, and detect molecular residual disease (MRD) after curative resection. Baseline ctDNA may help identify biologically aggressive tumors, early ctDNA clearance during neoadjuvant therapy can indicate favorable pathologic response, and postoperative ctDNA detection may reveal high-risk patients suitable for adjuvant therapy escalation or closer surveillance.

Recurrence after curative surgery remains the principal hurdle to durable survival in GEA. Contemporary peri-operative trials still show significant risk of recurrence often at a stage no longer amenable to salvage therapy [7,9,10,43,44,45]. Conventional surveillance with cross-sectional computed tomography (CT) ± carcinoembryonic antigen (CEA) and carbohydrate antigen 19-9 (CA19-9) misses sub-clinical disease, often attributed to imaging requiring a macroscopic tumor volume and serum markers lacking sensitivity [46,47]. ctDNA offers a biologically grounded alternative: fragments released from dying tumor clones are measurable in plasma within hours, so their persistence or re-emergence after treatment signals molecular residual disease, sometimes months before radiology [48]. Figure 2 summarizes the potential clinical workflow and timing of ctDNA assessment across the diagnostic, neoadjuvant, and postoperative settings in resectable GEA.

Tumor-informed multiplex-PCR assays provide some of the most mature evidence. In a real-world 11-center cohort of 295 stage I–III esophagogastric cancers, postoperative ctDNA was found in 23.5% of patients within 16 weeks of resection; 81.2% of these individuals relapsed vs. 13.5% of their ctDNA-negative counterparts, with a recurrence-free survival (RFS) hazard ratio (HR) of ~10.7 [95% confidence interval (CI) 4.3–29.3; *p* < 0.0001] [28]. Even more striking, ctDNA positivity at any time point postoperatively was associated with an 88.2% recurrence rate compared to 5.5% in ctDNA-negative patients, translating to a HR of 23.6 (95% CI 10.2–66.0; *p* < 0.0001) and a median RFS of 9.6 months vs. not reached. A subgroup analysis within the same cohort focused on 42 patients who achieved a pathologic complete or near-complete response (TRG 0–1) after neoadjuvant therapy, and still found that postoperative ctDNA positivity was associated with a sixfold increased risk of recurrence (RFS HR 6.2); notably, every patient with ctDNA positivity during surveillance eventually relapsed (HR 37.6), underscoring the prognostic power of ctDNA even in presumed clinically low-risk patients [49]. Emerging European data further support the tentative role of ctDNA while also introducing the feasibility of tumor-agnostic, methylation-based approaches. In a study of 86 patients with resectable gastric or gastroesophageal junction (GEJ) adenocarcinoma, a series of ctDNA testing was performed using TriMeth, a droplet digital PCR assay that identifies three gastrointestinal (GI) cancer-specific methylation markers [50]. ctDNA was detectable in 56% of patients prior to treatment [clinical tumor stage 3/4 (cT3/4) in 88.6%, and clinical node-positive (cN+) in 45.5%], but persisted in only 15% of patients at four weeks following surgical resection. Participants who remained ctDNA-positive at 4 weeks post-surgery experienced significantly worse outcomes, with a 24-month RFS of 12.5% (95% CI 2.0–78.2) compared to 70.7% (95% CI 58.4–85.5) in ctDNA-negative patients, and worse overall survival (OS) (24-month OS of ~20% vs. ~80% in ctDNA-negative patients; HR = 6.37, *p* = 0.001). Importantly, these observations remained significant in a multivariable analysis, independent of tumor stage or lymph node involvement. These data highlight that even without access to tumor-specific mutations, ctDNA-based MRD assessment using methylation signatures can identify high-risk patients and potentially guide personalized post-operative management.

Tumor-informed deep-sequencing studies in Asia add granularity. In a prospective study of 46 patients with stage I–III gastric cancer, ctDNA analysis was performed using a broad 1021-gene panel with serial plasma sampling [51]. Postoperative ctDNA was detectable in 18% of patients and was strongly associated with worse outcomes, with median disease-free survival (DFS) of 216 days versus not reached (DFS HR 6.56; OS HR 5.96), and all ctDNA-positive patients ultimately relapsing compared to 32% of ctDNA-negative patients (*p* = 0.0015). Longitudinal sampling further strengthened this association: ctDNA positivity at any postoperative time point conferred a DFS HR of 14.8 and preceded radiographic recurrence by a median of 179 days. In this cohort, ctDNA identified 84% of patients who relapsed and correctly classified 96% of those without recurrence, highlighting its accuracy and substantial lead-time advantage in identifying patients at highest risk of relapse following curative-intent surgery. Similarly, a prospective Chinese study evaluated 79 patients with stage II–III gastric cancer who received neoadjuvant chemotherapy followed by surgery and adjuvant chemotherapy [52]. ctDNA was analyzed at multiple time points using a high-depth hybrid-capture NGS assay targeting 425 cancer-related genes, with matched leukocyte DNA used to exclude germline and clonal hematopoiesis variants. Early molecular clearance after neoadjuvant therapy was associated with significantly improved survival, with a 3-year OS of 73% in ctDNA-negative patients vs. 34% in those with persistent ctDNA. Postoperative ctDNA positivity further stratified risk, with 3-year OS of 38% in ctDNA-positive patients compared to 68% in ctDNA-negative patients. Patients with persistently undetectable ctDNA after both neoadjuvant therapy and surgery had the most favorable outcomes, while conversion from negative to positive ctDNA after neoadjuvant therapy portended the worst survival.

A prospective analysis from the PLAGAST study evaluated 62 patients with locally advanced resectable gastric or GEJ adenocarcinoma using a tumor-informed 16-plex multiplex PCR assay (Signatera) to assess ctDNA at baseline, during neoadjuvant therapy, after neoadjuvant therapy, and in the postsurgical MRD window prior to adjuvant treatment [53]. Clearance of ctDNA during neoadjuvant therapy was associated with significantly improved outcomes, with a median RFS not reached vs. 13.3 months in patients with persistent ctDNA (HR 6.17; *p* = 0.002), and a 24-month OS rate of 95% vs. 59%. Post-NACT ctDNA positivity also predicted worse outcomes (mRFS 7.8 months vs. not reached; RFS HR 5.26; OS HR 7.35). In the MRD window, patients who remained ctDNA-positive had a median RFS of 3.6 months and OS of 8.6 months compared to not reached in ctDNA-negative patients (RFS HR 12.9; OS HR 14.5; both *p* < 0.0001). Patients who cleared ctDNA early had better 24-month RFS and OS rates than those who cleared late or remained positive. Persistent ctDNA positivity was also associated with poor pathologic response (TRG 4–5; *p* = 0.035). These results support the role of serial ctDNA monitoring to assess treatment response and identify patients at high risk of recurrence.

Preliminary results from a substudy of the phase III EXODOX trial (NCT04787354) also support the prognostic value of ctDNA in stage II–III gastric cancer [54]. Forty-two patients underwent tumor-informed MRD assessment using the CancerDetect™ (CeGaT GmbH, Tübingen, Germany.) assay at serial postoperative time points. Baseline ctDNA positivity was 45.2% and was more frequent in stage III than stage II disease (56.7% vs. 16.7%). ctDNA positivity at 6 and 12 months (29.6% and 35.2%) was significantly associated with inferior RFS, while baseline status alone was not. Dynamic changes in ctDNA further refined prognosis, as patients with persistent ctDNA positivity had the worst outcomes, whereas those who achieved ctDNA clearance after adjuvant chemotherapy had survival outcomes comparable to patients who remained negative throughout.

Evidence from a systematic review and meta-analysis of eight studies involving 423 patients supports these single-study findings [55]. Preoperative ctDNA positivity was associated with an increased risk of recurrence [relative risk (RR) 1.79; 95% CI 1.19–2.71], although individual studies demonstrated variability, likely due to limited sample sizes. Postoperative ctDNA detection conferred a stronger association with recurrence risk, with pooled risk ratios ranging from 3.17 to 3.68 depending on the model used. Across studies, ctDNA positivity was also associated with inferior RFS, with a pooled HR of 6.37 (95% CI 2.70–15.01). When stratified by timing, postoperative ctDNA positivity showed the highest prognostic value, correlating with a 14.09-fold increased risk of recurrence (95% CI 7.31–27.15). Similarly, ctDNA-positive status predicted worse OS, with pooled HRs of 4.58 (95% CI 1.68–12.49) for all time points and 11.78 (95% CI 4.40–31.53) for postoperative detection. These pooled data support ctDNA as a predictor of recurrence and survival across diverse cohorts and assay platforms, especially in the post-operative setting. Table 1 summarizes the major studies evaluating postoperative ctDNA-based MRD in resectable, including assay type, study design, and key outcomes.

### 3.2. MRD-Guided Management

The MRD-GATE trial is testing ctDNA-guided adjuvant chemotherapy in stage II–III gastric cancer after curative gastrectomy [56]. Interim results were presented at the recent ASCO annual meeting. Patients underwent tumor-informed ctDNA testing 28 days postoperatively and at regular intervals. MRD-negative patients received de-escalated therapy (observation for stage II, S-1 monotherapy for stage III) but could escalate to oxaliplatin-based doublets if they converted to MRD-positive. Baseline MRD-positive patients started with combination chemotherapy. Among 65 patients enrolled, 14 were MRD-positive and all received combination therapy. On the other hand, among 51 baseline-MRD negative patients, 45 received deescalated therapy at onset (9 received combination therapy after MRD conversion, and one refused) and six received combination therapy. The study is ongoing for follow-up and reporting the 3-year DFS (primary endpoint). However, it showed the feasibility of MRD-guided treatment, reducing adjuvant chemotherapy rate with acceptable 1-year DFS of 86.2%, and fewer toxicities.

Although not specific to gastric cancer, the CT002 trial evaluated ctDNA-guided adjuvant PD-1 blockade in resected dMMR solid tumors, including gastric/GEJ cancers. ctDNA was assessed 6–10 weeks after surgery. Patients who were ctDNA-positive received six months of pembrolizumab, while ctDNA-negative patients were observed. Early results presented at AACR 2025 showed that 85% (11/13) of ctDNA-positive patients treated with PD-1 blockade cleared ctDNA at six months, with a recurrence rate of 38%, compared with 100% recurrence in ctDNA-positive patients who did not receive immunotherapy. ctDNA-negative patients had favorable outcomes with observation alone, with a 2-year OS of 98% and recurrence in ~6%, supporting the safety of utilizing ctDNA to de-escalate therapy [57].

Several other trials are investigating MRD-based management with results still awaited. The CLAYMORE phase III trial in China is enrolling 416 patients with resected stage III gastric or GEJ adenocarcinoma, who are ctDNA-positive after surgery. Participants are randomized to receive adjuvant SOX chemotherapy with or without the PD-1 inhibitor tislelizumab. The trial will test whether adding immunotherapy improves DFS compared with chemotherapy alone. Results are awaited, but this is one of the first large randomized studies designed specifically for ctDNA-positive gastric cancer patients [58]. DECIPHER is a phase II trial conducted in the UK to evaluate trastuzumab deruxtecan (T-DXd) in operable HER2-positive GEA with detectable ctDNA after surgery and perioperative FLOT. The single-arm study uses Signatera for ctDNA detection and enrolls patients who have cleared standard multimodality therapy but remain ctDNA-positive. T-DXd is given for up to eight cycles, with the primary endpoint being ctDNA clearance after four cycles. Secondary endpoints include DFS, OS, quality of life, and safety. Recruitment is ongoing across multiple UK centers, with a planned sample size of 25 evaluable patients [59].

Interpretation of ctDNA-based MRD studies remains challenging due to methodological heterogeneity across cohorts. Variations in assay platforms (e.g., tumor-informed versus tumor-agnostic approaches), timing of blood collection relative to surgery or treatment, and thresholds used to define ctDNA positivity can substantially affect sensitivity and prognostic accuracy. These differences complicate direct comparison between studies and may partly explain the variability in reported recurrence risk estimates. Moreover, many available MRD studies are retrospective, single-center, and involve small patient populations, which may introduce selection and publication biases that overestimate the prognostic value of ctDNA. Prospective, standardized studies with harmonized methodologies and predefined endpoints are needed to validate ctDNA as a robust biomarker for MRD detection in gastroesophageal cancer.

Despite its strong prognostic correlations, there remain significant limitations when it comes to ctDNA-based MRD testing. The sensitivity of ctDNA appears to be suboptimal when measured at a single time point even with tumor-informed assays especially when tested too soon after surgery. Longitudinal testing, on the other hand, could improve sensitivity. However, given that the best timing to decide on adjuvant therapy is typically 4–8 weeks after the surgery; it remains unclear whether a one-time negative ctDNA result holds any clinical significance. Another question that remains unresolved is whether ctDNA is a reliable predictive factor to base the decision of escalating therapy on. In the case of colorectal cancer for instance, the ALTAIR trial tested adjuvant trifluridine/tipiracil (FTD/TPI) in patients with resected MRD-positive disease. The study did not meet its primary endpoint, with no statistically significant difference in DFS between FTD/TPI and placebo (median 9.3 vs. 5.6 months; HR 0.79, *p* = 0.107). This underscores that while ctDNA is a powerful prognostic tool, its role as a predictive biomarker of treatment benefit is not yet established. Therefore, caution should be taken when integrating ctDNA status into treatment decision making in GEA until we have more mature prospective evidence.

In addition, ctDNA detection is inherently limited in low-shedding GEA tumors, where the amount of tumor-derived DNA released into circulation may be minimal, leading to false-negative MRD results [60]. Although ctDNA clearance often correlates with improved outcomes, this finding remains prognostic rather than predictive, as there is no evidence that treatment modification based on ctDNA dynamics improves survival. Results from interventional studies, such as ALTAIR and early MRD-GATE, have not shown clear benefit from ctDNA-guided escalation, underscoring the need for randomized validation before such strategies are applied in clinical practice. Moreover, extrapolating MRD frameworks from colorectal or lung cancer to GEA should be performed with caution, as differences in tumor biology, histology, and shedding kinetics may affect assay performance and clinical interpretation. The detectability of ctDNA can also be influenced by several biologic and clinicopathologic factors, including tumor histology, differentiation, stage, and metastatic pattern. These determinants may partly explain interstudy variability in ctDNA yield and assay sensitivity. Table 2 summarizes representative studies exploring how these features impact ctDNA shedding and detection in GEA.

## 4. ctDNA in Advanced/Metastatic GEA

### 4.1. Baseline Prognostic Value

**Figure 3:**
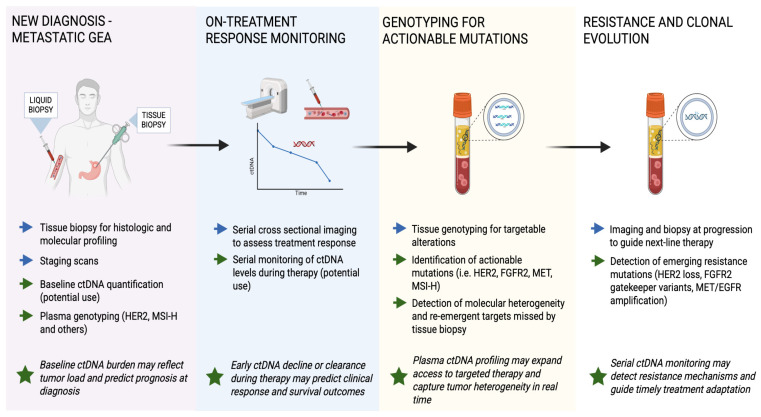
Clinical workflow of ctDNA applications in metastatic gastroesophageal adenocarcinoma (GEA). Blue arrows indicate standard management steps, while green arrows represent ctDNA-based applications across diagnostic, therapeutic, and resistance settings. Green stars highlight key clinical implications. In metastatic GEA, ctDNA serves multiple roles: establishing baseline molecular profiles, monitoring treatment response, identifying actionable mutations for targeted therapy, and detecting emerging resistance before radiographic progression. Serial ctDNA monitoring provides a real-time, non-invasive view of tumor dynamics and may guide therapy adaptation or sequencing in advanced disease.

Similarly to the early-stage setting, multiple studies have shown the prognostic utility of ctDNA in metastatic GEA patients. In the prospective LIQUID BIO study, investigators enrolled 72 patients with metastatic GEA and used a tumor-informed amplicon NGS assay that tracked up to 48 patient specific single nucleotide variants [64]. ctDNA was detectable in 75% of cases, and patients with two or more tracked mutations at baseline had considerably worse outcomes, with median progression-free survival (PFS) of 4.5 months vs. 9.2 months (*p*-value = 0.009) and OS of 8.4 months vs. 16.5 months (*p*-value = 0.014). Meanwhile, real-world data from the GuardantINFORM registry, which included 824 US patients with advanced gastric cancer profiled via the tumor-agnostic Guardant360 hybrid-capture NGS assay (73 genes), showed that a baseline maximum variant allele frequency (VAF) > 2.9% predicted an earlier need for therapy change (4.8 vs. 7.4 months, *p*-value < 0.001) and shorter OS (13.2 vs. 19.1 months, *p*-value < 0.001) even after adjusting for standard clinical factors such as Eastern Cooperative Oncology Group (ECOG) status and metastatic burden [65]. A meta-analysis of 22 ctDNA studies, predominantly using tumor-agnostic ddPCR or ≤50 gene NGS panels in 1144 metastatic GEA, found that ctDNA positivity at baseline carried a pooled HR for death of 3.87 [66]. Despite methodological differences, these studies consistently demonstrate that higher ctDNA burden at diagnosis is associated with poorer prognosis in metastatic GEA. Figure 3 illustrates how ctDNA is integrated across the disease course in metastatic GEA, from diagnosis and treatment monitoring to detection of actionable mutations and resistance mechanisms.

### 4.2. On-Treatment Response Monitoring

Beyond prognostic potential, ctDNA has been explored as a tool for dynamic response monitoring during therapy. The phase II PRODIGE 59 DURIGAST trial involved 97 heavily pre-treated patients with advanced GEA and used tumor-agnostic methylation-based ddPCR (TriMeth) to assess ctDNA changes at baseline and week 4 of therapy [67]. Patients experiencing a ≥75% drop in methylation signal had markedly better outcomes, objective response rate (ORR) 55%, median PFS 7.4 months, and OS 16.0 months, compared to those without such decline, who had an ORR of 16%, PFS 2.2 months, and OS 6.6 months (*p* < 0.05 for the three endpoints). In a separate cohort of 30 chemotherapy-naïve metastatic gastric cancer patients treated with pembrolizumab plus capecitabine/oxaliplatin, a tumor-agnostic 425-gene hybrid-capture NGS assay was used to assess plasma ctDNA [68]. Clearance of baseline ctDNA by cycle 2 (achieved in 8 patients) was associated with significantly improved outcomes, ORR 83% and median PFS 15.6 months, compared to persistent ctDNA (*n* = 22), where ORR was 29% and median PFS was only 6.0 months (*p* < 0.05 for both endpoints). While neither study incorporated ctDNA to guide treatment decisions prospectively, their results strongly support the potential of early ctDNA dynamics to reflect therapeutic efficacy, with implications for future interventional trials.

### 4.3. Plasma Genotyping for Actionable Targets

ctDNA-based genotyping has shown substantial value in identifying actionable molecular targets, especially when tumor tissue is insufficient or inaccessible. In a large retrospective analysis of 1630 metastatic GEA patients using the tumor-agnostic Guardant360 hybrid-capture panel, Maron and colleagues identified key actionable alterations: HER2 amplified in 9.5%, *FGFR2* in 7.7%, MET proto-oncogene, receptor tyrosine kinase (MET) in 5.6%, epidermal growth factor receptor (*EGFR*) in 4.9%, and MSI-H in 3.2% [29]. Notably, in the subgroup whose HER2 amplification was detected only in plasma, and who subsequently received trastuzumab, the median OS reached 26.3 months, compared with just 7.4 months for plasma-positive patients who did not receive HER2-targeted therapy, highlighting how ctDNA can meaningfully extend therapeutic access (*p* = 0.002). Similarly, Schrock et al. studied 160 patients with metastatic GEA who underwent matched tissue and plasma genotyping using the same tumor-agnostic hybrid-capture NGS platform (FoundationOne for tissue, Guardant360 for plasma) [69]. Plasma ctDNA recapitulated more than 80% of tissue-detected driver alterations and uncovered additional, potentially actionable mutations in approximately one-third of patients, demonstrating ctDNA’s ability to capture spatial and clonal tumor heterogeneity beyond what is seen in a single-site tissue biopsy. Real-world data also support the clinical utility of plasma-based HER2 detection to guide therapy in advanced GEA [70]. In a GuardantINFORM analysis, 215 patients with *ERBB2* amplification detected by ctDNA were identified, of whom 135 (63%) received HER2-directed therapy. Patients treated based on ctDNA findings had significantly longer real-world time to treatment discontinuation (rwTTD 5.8 vs. 1.9 months; HR 0.47, 95% CI 0.34–0.65, *p* < 0.01) and real-world time to next treatment (rwTTNT 9.4 vs. 6.3 months; HR 0.55, 95% CI 0.37–0.81, *p* < 0.01), although no statistically significant difference in real-world OS was observed (not reached vs. 22 months; HR 0.67, 95% CI 0.41–1.08, *p* = 0.10). These findings suggest that ctDNA-identified *ERBB2* amplification can be used to expand access to HER2-directed therapy by identifying patients who retain or reacquire HER2 amplification and could benefit from continued or reintroduced HER2-targeted therapy. Results from an exploratory analysis based on the phase II DESTINY-Gastric01 trial further highlighted ctDNA as a marker in refining patient selection for T-DXd [71]. In the primary cohort of HER2-positive gastric cancer, plasma *HER2* amplification detected by ctDNA correlated with higher ORR (61% vs. 34% in patients without amplification). Patients with high adjusted plasma copy number (apCN ≥ 18.2) of HER2 had favorable outcomes, with ORR approaching 79% and median OS of 16.6 months compared to 8.6 months in those with low apCN. On the other hand, detection of co-amplifications in *MET*, *EGFR*, or *FGFR2* using ctDNA was associated with lower ORR, potentially pointing towards mechanisms of resistance. These results highlight how liquid biopsy-based HER2 quantification could augment tissue testing by capturing spatial heterogeneity and help identify patients likely to benefit from HER2-targeted therapy.

Loss of HER2 expression following trastuzumab-based therapy is increasingly recognized as a major clinical challenge in metastatic GEA. The prospective GASTHER3 study evaluated 48 patients with initially HER2-positive gastric cancer who underwent paired biopsies before and after first-line trastuzumab-containing chemotherapy [72]. Nearly one-third (29%) of patients lost HER2 positivity on post-progression biopsy, accompanied by a significant decline in median H-score (from 225 to 175, *p* = 0.047) and an increase in HER2 genetic heterogeneity (from 2.9% to 21.9%). Among those who received second-line trastuzumab emtansine, patients with persistent HER2 amplification had an ORR of 44%, whereas none of the patients who lost HER2 expression responded, suggesting that loss of HER2 may drive resistance to subsequent anti-HER2 therapy. These data underscore the dynamic nature of HER2 expression under therapeutic pressure. In this context, ctDNA testing offers a practical and less invasive alternative to repeat biopsies for reassessing HER2 status as shown in the GuardantINFORM study.

Similarly to HER2, other biomarkers and gene alterations can be detected in ctDNA and can potentially guide treatment. In 2022, Jogo et al. applied a tumor-agnostic 300 gene hybrid-capture NGS panel to 365 Japanese patients with metastatic gastric cancer [73]. They detected *FGFR2* amplification in 7.7% of plasma samples compared to just ~3% in matched tissue, with two plasma positive/tissue negative patients responding to the *FGFR2* inhibitor, futibatinib. The PANGEA trial tested a biomarker-driven treatment algorithm in metastatic GEA using serial tissue and plasma profiling [74]. Sixty-eight patients were enrolled, with a 1-year OS of 66% and a median OS of 15.7 months, superior to historical controls. Notably, one-third of patients showed discordance between primary and metastatic tissue at baseline, and nearly half of the patients showed a change in their biomarker group at the time of progression. Of note, two patients with FGFR2 amplification were treated with bemarituzumab plus mFOLFOX6 with initial benefit; however, both patients progressed and were eventually reassigned to different groups, illustrating resistance development. In RTK-amplified tumors more broadly, progression was often accompanied by loss of amplification or acquisition of MAPK/PI3K alterations, and in some cases PD-L1 upregulation. These studies underscore ctDNA’s relevance not only in highlighting known targets but in identifying otherwise undetected mutations that can influence treatment.

While these findings highlight the expanding role of ctDNA in identifying actionable alterations, several interpretive challenges remain. Analytical concordance between ctDNA and tissue genotyping varies by target and assay, typically ranging from 70 to 90% for amplifications such as HER2 or *FGFR2* [75]. However, thresholds for calling amplification events in plasma are not standardized, with most platforms relying on assay-specific copy number or allele frequency cutoffs that may underdetect low-level amplifications. Detection rates also correlate with tumor burden, site of metastasis, and prior systemic therapy, with patients who have liver-dominant or high-volume disease generally exhibiting higher ctDNA yield [76]. Importantly, these associations are largely retrospective, and plasma-detected targets still require validation in prospective biomarker-selected trials before guiding routine treatment [77]. Overall, while ctDNA offers a valuable complement to tissue profiling, its therapeutic implications remain hypothesis-generating rather than practice-changing until supported by randomized data.

### 4.4. Resistance and Clonal Evolution

In addition to tracking response and guiding therapy, ctDNA has also demonstrated promise for early detection of resistance mechanisms before radiographic progression. This application may be particularly useful in tailoring salvage therapy strategies. In the PRODIGE 59 DURIGAST trial, 97 patients with metastatic HER2-positive gastric or GEJ adenocarcinoma underwent serial ctDNA monitoring using a tumor-agnostic methylation-based ddPCR assay (TriMeth) and amplicon-based NGS [67]. Among 37 patients with plasma available at progression, emergent resistance alterations, *MET* amplifications (*n* = 4), *EGFR* amplifications (*n* = 2), and secondary HER2 mutations (*n* = 3) were detected in ctDNA but absent at baseline, highlighting its utility for early resistance detection. Furthermore, patients with ≥75% decline in methylation signal at week 4 had improved outcomes: ORR 55%, PFS 7.4 months, and OS 16.0 months, versus ORR 16%, PFS 2.2 months, and OS 6.6 months in those without early clearance (*p* < 0.05 for the three endpoints). Jogo et al.’s *FGFR2* study further reinforced this concept: gatekeeper N549H/K mutations, linked to resistance to *FGFR2* blockade, emerged in plasma ctDNA 4 to 12 weeks before radiologic progression, underscoring ctDNA’s ability to anticipate relapse [73]. These findings collectively advocate for ctDNA as a non-invasive tool to assess clonal evolution and resistance mechanisms. For example, loss of HER2 amplification or the appearance of *FGFR2* gatekeeper mutations may signal resistance and prompt a switch to another targeted or combination therapy. Ongoing ctDNA monitoring could also help track how well these adjustments are working, supporting a more flexible and individualized approach to managing advanced GEA.

### 4.5. Implementation, Feasibility and Limitations

On a practical level, the integration of ctDNA testing into oncology workflows is becoming increasingly feasible. ctDNA testing reduced the median time to clinical trial enrollment from 33 days to 11 days (*p*-value < 0.0001) [78]. These findings highlight the potential of ctDNA to expedite patient access to biomarker-guided therapies, though broader adoption will require improvements in standardization, cost-efficiency, and clinician training. However, pre-analytical and analytical variability remain major barriers to consistency and reproducibility. Differences in plasma processing time, ctDNA extraction methods, and storage conditions can affect yield and fragment integrity, while variation in library preparation and variant-calling algorithms contributes to inconsistent detection sensitivity. The absence of standardized cutoffs for variant allele frequency or copy-number gain, as well as differences in reporting formats, complicates cross-study comparison [79]. International initiatives such as the BloodPAC Consortium and the ESMO Translational Research Working Group are developing frameworks to harmonize assay validation and quality control, but universal standards are still lacking [80,81].

While ctDNA testing continues to expand, its cost and accessibility remain major barriers to global implementation. Tumor-informed assays require sequencing of both the tumor and multiple plasma samples, with per-test costs often exceeding $3000 USD in the United States. Such expenses make routine use difficult, particularly in low- and middle-income countries where gastric and esophageal cancers are most prevalent [82]. Simpler methylation-based approaches, such as TriMeth, may offer more affordable alternatives, as they avoid individualized tumor sequencing. For ctDNA testing to achieve broader impact, improving affordability and access will be as important as refining technical performance.

While multiple studies have evaluated ctDNA in metastatic or advanced GEA, comparing results across them is challenging due to methodological differences. Some studies used tumor-informed multiplex PCR assays with high specificity for known variants, whereas others applied tumor-agnostic hybrid-capture NGS or methylation-based approaches, which provide broader coverage but lower sensitivity. These variations influence ctDNA detection rates, the interpretation of VAF, and the generalizability of reported outcomes.

Beyond methodological variability, ctDNA interpretation can be complicated by biological and technical factors. Discordance between tissue and plasma genotyping may arise from sampling at different time points or intratumoral heterogeneity, while clonal hematopoiesis of indeterminate potential (CHIP) can lead to false-positive results by introducing non-tumor variants into plasma DNA [83]. Recognizing these limitations is critical to ensure accurate interpretation and clinical decision making.

Commercial ctDNA assays such as Guardant360, FoundationOne Liquid, and Signatera have facilitated broader clinical use but also introduce important considerations. Each platform differs in design, ranging from tumor-informed to tumor-agnostic approaches, and may vary in analytical sensitivity, genomic coverage, and reporting thresholds. These differences, along with potential conflicts of interest and limited cross-platform validation, can influence reported performance metrics and complicate data interpretation. Independent, head-to-head evaluations are needed to standardize assay performance and ensure that clinical adoption is guided by objective evidence rather than proprietary variability.

Currently, ctDNA should be viewed as a valuable complement to imaging and tissue biopsy in the care of patients with metastatic GEA, with ongoing trials expected to define its role more clearly in the years ahead. Table 3 summarizes all ctDNA applications across MRD, prognosis, therapy monitoring, detecting actionable alterations, and resistance.

## 5. Conclusions

ctDNA emerged as a powerful adjunctive tool in GEA, offering real-time molecular insights that extend beyond traditional imaging and tissue biopsy. Despite these advances, ctDNA remains primarily a prognostic rather than predictive biomarker. Its optimal timing, assay platform, and interpretation thresholds are not yet standardized, and low-shedding tumors and clonal hematopoiesis continue to limit sensitivity and specificity. Ongoing randomized and biomarker-guided trials will determine whether ctDNA-directed escalation or de-escalation improves outcomes.

Future research should focus on harmonizing assay methodologies, establishing validated thresholds for ctDNA detection, and determining whether ctDNA-guided escalation or de-escalation strategies translate into survival benefit. Equally important will be addressing cost and access barriers, particularly in regions where GEA burden is highest. With robust standardization, prospective validation, and integration into biomarker-driven clinical trials, ctDNA holds the potential to transform precision management in GEA by refining risk stratification, guiding adjuvant decisions, and enabling real-time therapeutic monitoring. For now, ctDNA should be viewed as complementary—not substitutive—to imaging and tissue biopsy.

## Figures and Tables

**Table 1:** Studies evaluating postoperative ctDNA-based MRD in resectable GEA.

Author/Study Design	Number of Subjects (Characteristics)	Assay Type	Major Study Findings	Median Lead Time to Recurrence
Huffman et al. [28]/retrospective cohort	295 (stage I–III; 68 in MRD window)	Tumor-informed 16-plex mPCR-NGS (Signatera)	Postoperative ctDNA (MRD) was detected in 23.5%; recurrence in 81.2% vs. 13.5% (RFS HR 10.7). Anytime postoperative ctDNA was detected in 27.2%; recurrence in 88.2% vs. 5.5% (RFS HR 23.6)	Not reported
Lander et al. [49]/retrospective cohort	42 (23 in MRD window; pCR/near-pCR subset)	Tumor-informed 16-plex mPCR-NGS (Signatera)	Postoperative ctDNA (MRD) detected in 13%; recurrence in 67% vs. 15% (RFS HR 6.2). ctDNA positivity during surveillance detected in 15.6%; recurrence in 100% vs. 7.4% (RFS HR 37.6).	78 days
Iden et al. [50]/prospective, observational cohort study	86 (53 with post-op samples)	Tumor-agnostic methylation ddPCR (TriMeth)	Postoperative ctDNA detected in 15%; 24-month RFS 12.5% vs. 70.7% and OS ~20% vs. ~80% (HR 6.37, *p* = 0.001).	Not reported
Yang et al. [51]/prospective, observational cohort study	46	Tumor-informed 1021-gene hybrid-capture NGS panel (1.09 Mb)	Postoperative ctDNA (MRD) detected in 18%; recurrence in 100% vs. 32% (DFS HR 6.56, *p* < 0.0001; OS HR 5.96, *p* = 0.0007). ctDNA positivity at any postoperative time point detected in 84% of patients who recurred vs. 4% without recurrence (*p* < 0.0001). Sensitivity was 39% and specificity was 100%, for predicting recurrence at 30 months.	179 days
Zhang et al. [52]/prospective, observational cohort study	79 (57 underwent surgery)	Tumor-informed high-depth hybrid-capture NGS (425 genes)	Postoperative ctDNA-positive 3-yr OS 38% vs. 68% in ctDNA-negative. After neoadjuvant chemotherapy (NACT): 34% vs. 73%. Persistent ctDNA negativity after NACT/surgery associated with longest OS; negative to positive conversion after NACT predicted worst survival.	Not reported
Zaanan et al. [53]/prospective, observational cohort study	62 (50 evaluable MRD)	Tumor-informed mPCR-NGS (Signatera)	Postoperative ctDNA (MRD) positivity associated with markedly worse outcomes; RFS HR 12.94 (median RFS 3.57 mo vs. NR) and OS HR 14.54 (median OS 8.59 mo vs. NR).	184 days
Han et al./Prospective phase III randomized trial sub-study, interim results (EXODOX, NCT04787354) [54]	42	Tumor-informed bespoke WES-based panel (CancerDetect™ (CeGaT GmbH, Tübingen, Germany), IMBdx (IMBdx Inc., Seoul, Republic of Korea))	Postoperative ctDNA positivity at 3–10 weeks (P0) 45.2%; not significantly associated with RFS (P = 0.101). ctDNA positivity at 6 months (P1 = 29.6%) and 12 months (P2 = 35.2%) predicted worse RFS (P = 0.003 and 0.038). Persistent ctDNA positivity post-ACT conferred poorest RFS, whereas ctDNA clearance after ACT yielded RFS comparable to persistently negative patients (P = 0.480).	Not reported

ACT = adjuvant chemotherapy; ctDNA = circulating tumor DNA; DFS = disease-free survival; HR = hazard ratio; MRD = molecular residual disease; NACT = neoadjuvant chemotherapy; NGS = next-generation sequencing; NR = not reached; OS = overall survival; RFS = recurrence-free survival; TRG = tumor regression grade; WES = whole-exome sequencing.

**Table 2:** Biologic and clinicopathologic factors influencing ctDNA detectability in GEA.

Characteristic	Subgroup(s)	Representative Studies	Assay Type/Setting	ctDNA Detection Findings	Interpretation/Comment
Histology	Diffuse/Signet-ring vs. Intestinal/Non-diffuse	Leal A et al. [61]	Tumor-agnostic hybrid-capture	Median mutant allele fraction (MAF) significantly higher in intestinal than in diffuse gastric cancers (*p* = 0.02).	Diffuse-type tumors exhibited lower ctDNA fraction, consistent with lower shedding and plasma detectability
Tumor differentiation	Well, moderate, poor	Leal A et al. [61]	Tumor-agnostic hybrid-capture	Trend toward higher MAF in poorly differentiated tumors (*p* = 0.07, NS).	Poorly differentiated tumors may shed more ctDNA due to greater cellular turnover and necrosis, though the association was not statistically significant.
Disease Stage	Stage I vs. Stage II–III	Zaanan A et al. [53]	tumor-informed (Signatera)	Baseline ctDNA detection: 37.5% in Stage I (*n* = 16), 81% in Stage II (*n* = 21), 87.5% in Stage III (*n* = 16).	ctDNA detectability rises with advancing stage suggesting different shedding biology where small, early-stage (T1–2N0) tumors release insufficient ctDNA for reliable detection.
Primary vs Metastatic Site	Peritoneal-only vs. Visceral/Liver	Sullivan BG et al. [62]; Ococks E et al. [63]	Tumor-agnostic hybrid-capture (Guardant360); tumor-informed (Signatera)	Significantly lower mean mVAF was observed in peritoneal carcinomatosis-only group compared with patients with visceral metastases (14.2 ± 42 vs. 36.7 ± 56.5; *p* < 0.01). Ococks et al. reported one recurrence confined to peritoneum that was missed by Signatera.	Peritoneal involvement yields reduced plasma ctDNA levels; potentially limiting surveillance advantage in this group

ctDNA: circulating tumor DNA; GEA: gastroesophageal adenocarcinoma; MAF: mutant allele fraction; mVAF: mean variant allele fraction; NS: not significant; NAT: neoadjuvant therapy.

**Table 3:** Clinical Applications and Limitations of ctDNA in Gastroesophageal Adenocarcinoma and Their Implications.

Application	Clinical Utility	Limitations
Minimal Residual Disease (MRD)	Predicts relapse risk	Prognostic but not predictive
Monitoring	Real-time treatment efficacy	Unclear thresholds for “molecular response”
Actionable Alterations	Expands therapy access (HER2, MSI-H)	May miss low-shedding tumors
Resistance	Detects new drivers of progression	Still investigational

ctDNA: circulating tumor deoxyribonucleic acid, HER2: human epidermal growth factor receptor 2, MRD: minimal residual disease, MSI-H: microsatellite instability-high.
